# Advanced Cellular Models for Neurodegenerative Diseases and PFAS-Related Environmental Risks

**DOI:** 10.3390/neurosci6040125

**Published:** 2025-12-08

**Authors:** Davide Rotondo, Laura Lagostena, Valeria Magnelli, Francesco Dondero

**Affiliations:** 1 Department of Science and Technological Innovation, Università del Piemonte Orientale, 15121 Alessandria, Italy; davide.rotondo@uniupo.it (D.R.); valeria.magnelli@uniupo.it (V.M.); 2 Institute of Biophysics, National Research Council, Via de Marini 6, 16149 Genova, Italy; laura.lagostena@ibf.cnr.it

**Keywords:** gene–environment interactions, induced pluripotent stem cells (iPSC), microphysiological systems, neurotoxicity, per- and polyfluoroalkyl substances (PFAS)

## Abstract

Per- and polyfluoroalkyl substances are persistent environmental contaminants increasingly implicated in neurotoxicity. Establishing causality and mechanisms relevant to Alzheimer’s disease, Parkinson’s disease, and multiple sclerosis requires human-relevant systems that capture exposure, barrier function, and brain circuitry. We review advanced cellular platforms—iPSC-derived neuronal and glial cultures, cerebral and midbrain organoids, and chip-based microphysiological systems—that model disease-relevant phenotypes (Aβ/tau pathology, dopaminergic vulnerability, myelination defects) under controlled PFAS exposures and defined genetic risk backgrounds. Modular, fluidically coupled BBB-on-chip → brain-organoid microphysiological systems have been reported, enabling chronic, low-dose PFAS perfusion under physiological shear, real-time barrier integrity readouts such as transepithelial/transendothelial electrical resistance (TEER), quantification of PFAS partitioning and translocation, and downstream neuronal–glial responses assessed by electrophysiology and multi-omics. Across platforms, convergent PFAS-responsive processes emerge—mitochondrial dysfunction and oxidative stress, lipid/ceramide dysregulation, neuroinflammatory signaling, and synaptic/network impairments—providing a mechanistic scaffold for biomarker discovery and gene–environment interrogation with isogenic lines. We outline principles for exposure design (environmentally relevant ranges, longitudinal paradigms), multimodal endpoints (omics, electrophysiology, imaging), and cross-lab standardization to improve comparability. Together, these models advance the quantitative evaluation of PFAS neurotoxicity and support translation into risk assessment and therapeutic strategies.

## 1. Introduction

Per- and polyfluoroalkyl substances (PFAS) are a class of highly persistent industrial chemicals that have become ubiquitous environmental contaminants due to their extensive use in consumer products and industrial applications. Their remarkable stability and bioaccumulative properties raise significant concerns for human health, as PFAS have been detected in water, soil, wildlife, and human tissues worldwide [1,2]. Epidemiological studies associate PFAS exposure with a spectrum of adverse outcomes, including metabolic dysfunction, immunotoxicity, and neurodevelopmental deficits [3,4]. Notably, however, individual responses to PFAS such as uptake and bioaccumulation vary widely, suggesting that genetic factors may modulate susceptibility to PFAS toxicity.

Gene–environment interaction (G × E) refers to the phenomenon whereby the effect of an environmental exposure on disease risk depends on an individual’s genotype, and conversely, genetic influences manifest differently depending on environmental context [5]. In the case of PFAS, emerging data indicate that polymorphisms in genes involved in lipid metabolism (e.g., PPARα/γ), hormone signaling, and xenobiotic clearance may alter the biological impact of a given PFAS concentration [6]. Yet, to date, although inter-individual genetic variability likely modulates susceptibility, the underlying mechanisms remain unresolved because PFAS-focused genome-wide association or interaction studies have not yet been undertaken.

Emerging evidence indicates that PFAS can affect the central nervous system, potentially contributing to neurodegenerative processes [3]. This perspective aims to bridge that gap by providing a review of human-relevant cellular models, such as induced pluripotent stem cells (iPSC)-derived neurons, midbrain and cerebral organoids, and microphysiological systems, and their use in dissecting the mechanisms by which PFAS contributes to Alzheimer’s disease (AD), Parkinson’s disease (PD), and multiple sclerosis (MS). We highlight how these advanced three-dimensional (3D) platforms enable controlled PFAS exposures in defined genetic backgrounds (e.g., *APOE ε4*, *LRRK2*, HLA-DRB1*15:01) to reveal G × E interactions at molecular, cellular, and functional levels. This perspective synthesises evidence across transcriptomics, lipidomics, immunoassays and electrophysiology to outline a mechanistic framework for PFAS neurotoxicity, and it introduces a pragmatic roadmap for interrogating gene–environment (G × E) interactions in advanced human models.

### Environmental Pollutants and Neurodegeneration

Neurodegenerative disorders such as Alzheimer’s disease (AD), Parkinson’s disease (PD), and multiple sclerosis (MS) are complex conditions with multifactorial etiologies involving both genetic predispositions and environmental exposures. This reflects a growing recognition that environmental pollutants, especially “forever chemicals” like per- and polyfluoroalkyl substances (PFAS), may contribute to the onset or progression of these diseases. At the same time, each disease has well-established genetic and genomic associations that influence susceptibility (e.g., *APOE ε4* in AD, *LRRK2* in PD, *HLA* variants in MS). To disentangle these factors and study disease mechanisms, researchers are turning to advanced physiological models. In particular, advanced human cellular models provide a relevant platform for dissecting gene–environment interactions in neural tissue [7,8]. This report provides a detailed literature analysis of the state-of-the-art in vitro models for AD, PD, and MS, highlighting the best cellular/organotypic systems (e.g., organoids and 3D cultures) used to study these diseases. Importantly, we focus on evidence linking pollutants like PFAS to neuropathology, while also considering how genetic and genomic factors can be incorporated into these models.

Associations between environmental pollutants and neurodegenerative disorders are not new. For example, exposure to heavy metals such as lead and mercury has been associated with increased Alzheimer’s risk [9], while pesticide exposure, particularly to paraquat and rotenone, correlates with elevated Parkinson’s incidence [10]. Chronic exposure to fine particulate air pollution has also been linked to accelerated cognitive decline and higher dementia risk [11,12].

Human epidemiological studies link higher serum per- and polyfluoroalkyl substances levels to increased all-cause, Alzheimer’s disease, and Parkinson’s disease (PD) mortality [3], while animal and in vitro models demonstrate PFAS-induced dopaminergic neuron loss and heightened glutamatergic signaling [13]. PFAS, particularly perfluorooctanoic acid (PFOA) and perfluorooctane sulfonate (PFOS), cross the blood–brain barrier (BBB), accumulating in neural tissues [2,14], and BBB dysfunction itself has been shown to exacerbate AD pathology [15].

Ceramide accumulation acts as a potent neuroinflammatory signal by activating NF-κB–driven cytokine expression, promoting NLRP3 inflammasome assembly in microglia, and perturbing membrane microdomains to sensitize TLR4 signaling. It also compromises mitochondrial integrity, releasing reactive oxygen species and mitochondrial DNA that further stimulate glial immune responses [16,17,18,19]. Transcriptomic and lipidomic analysis of PFOS- and PFOA-exposed SH-SY5Y neurons revealed coordinated upregulation of inflammatory mediators (IL-1β, TNF-α) and dysregulated sphingolipid metabolism, findings that align with ceramide-driven neuroinflammatory pathways [20]. These pathways underscore how PFAS-driven ceramide accumulation can directly trigger neuroinflammatory signaling, amplifying neuronal damage in AD, PD, and MS models.

Mechanistic toxicology studies further show that PFAS can disrupt neurotransmission pathways in the brain, with consistently decreased dopaminergic activity and suppressed GABA_A receptor–mediated currents observed after PFAS exposure [13,21]. Additionally, PFAS induce oxidative stress, calcium dyshomeostasis, and neuroinflammation, promoting neurodegeneration [14]. This mechanistic framework is further supported by studies demonstrating that PFOS exposure selectively impairs dopaminergic neuron viability and triggers neuroinflammatory cascades in both animal models and cellular systems [14].

**Figure 1:**
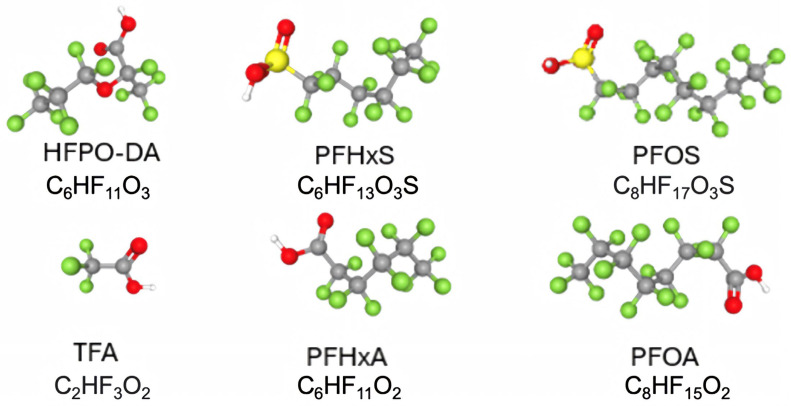
Representative chemical structures of selected per- and polyfluoroalkyl substances (PFAS)PFAS are a multi-class family that includes perfluoroalkyl carboxylates, sulfonates, ether derivatives, and ultra-short-chain species. Shown are legacy long-chain PFAS listed under the Stockholm Convention—perfluorooctanoic acid (PFOA), perfluorooctane sulfonic acid (PFOS), and perfluorohexane sulfonic acid (PFHxS)—together with short-chain replacements perfluorohexanoic acid (PFHxA) and hexafluoropropylene oxide dimer acid (HFPO-DA, “GenX”), and the ultra-short-chain PFAS trifluoroacetic acid (TFA), a pervasive terminal degradation product of multiple fluorinated substances (including some hydrofluoroolefins and pharmaceuticals). Molecular formulas are shown beneath each structure. Atom colors in the ball-and-stick models: carbon atoms are shown in grey, fluorine in green, oxygen in red, sulfur in yellow, and hydrogen in white.

Given these concerns, scientists are leveraging advanced in vitro models to directly test how pollutants affect human neural cells and to probe gene–environment interactions. Traditional animal models often fail to capture human genetic nuances and chronic exposures [22]. Human cellular models, especially 3D organoids, provide a powerful alternative, as they approximate aspects of human brain development and microarchitecture while carrying patient-specific genetics and allowing controlled exposures [8]. Given their widespread use, persistence, and potential to accumulate in biological systems, a subset of PFAS has become the primary focus of toxicological and mechanistic studies (Figure 1).

## 2. Alzheimer’s Disease: Organoids and 3D Cell Models

Alzheimer’s disease (AD) is characterized by amyloid-β (Aβ) plaques, tau tangles, synaptic loss, and neuroinflammation. Amyloid-β (Aβ) peptides are generated from amyloid precursor protein (APP) by sequential β- and γ-secretase cleavage, producing mainly Aβ_1–40_ and the more aggregation-prone Aβ_1–42_ isoform. At low concentrations, monomeric Aβ supports synaptic plasticity and neuronal survival by modulating long-term potentiation [23]. However, when production exceeds clearance or when clearance is impaired, as in *APOE ε4* carriers, Aβ_1–42_ rapidly self-associates into soluble oligomers, protofibrils, and insoluble fibrils which form plaques starting from the prefrontal cortex. Moreover, in familial forms of AD, mutations in presenilin (PSEN1/PSEN2), the catalytic subunit of the γ-secretase complex, lead to impaired Aβ processing and greater accumulation of the aggregation-prone Aβ_1–42_, illustrating how genetic predisposition can set the stage upon which environmental stressors further accelerate pathology [24,25]. Electrophysiological approaches using multielectrode arrays have shown that Aβ can disrupt neuronal firing and network synchronization, providing functional evidence of early synaptic impairment relevant for PFAS-driven neurotoxicity [26]. Over time this accumulation leads to phosphorylation of tau protein (p-tau) inside the neuron and its aggregation in intraneuronal neurofibrillary tangles (NFTs). Consequently, the patient develops short-term memory problems followed by severe cognitive impairments, loss of anterograde and retrograde memory and of the normal behaviour. These oligomeric species disrupt N-methyl-D-aspartate (NMDA) and other synaptic receptors, impair mitochondrial function, alter calcium homeostasis, and activate microglia, leading to neuroinflammation [27].

Quantification of Aβ and p-tau in both clinical and experimental settings employs sensitive biochemical and imaging techniques. Soluble and insoluble Aβ levels are measured by ELISA or mass spectrometry in cerebrospinal fluid and brain tissue, often reporting reduced Aβ_42_/Aβ_40_ ratios as a disease biomarker [28,29]. In organoid and cellular models, Aβ deposits are visualized by immunostaining with antibodies such as 6E10 and by Thioflavin-S labeling of fibrillar aggregates. p-tau pathology is assessed by detecting hyperphosphorylated tau species (e.g., p-tau181, p-tau217) using ultrasensitive immunoassays [30] and by Western blot or immunofluorescence analyses in cell and organoid cultures [31,32].

Patient-derived cerebral organoids have become invaluable in modeling Alzheimer’s pathology, with in-depth frameworks now available to recapitulate disease hallmarks such as Aβ and p-tau accumulation [33]. iPSC-derived organoids also recapitulate key AD pathologies [34]. Similarly, 3D human brain-like tissue constructs have demonstrated that viral challenge is sufficient to induce Aβ accumulation and neuronal loss, highlighting how environmental exposures, including PFAS, may act as upstream triggers of Alzheimer’s pathology [35]. Raja et al. (2016) [32] generated organoids with familial AD mutations that spontaneously formed Aβ plaques and tau tangles in 3D culture.

Lu et al. (2024) [31] using human cerebral organoids derived from healthy donor iPSCs and dual-SMAD inhibition, a differentiation strategy that blocks BMP and TGF-β signaling to efficiently direct stem cells toward a neural fate, demonstrated that chronic exposure to PFOA, PFOS, and perfluorohexane sulfonate (PFHxS) significantly increased both soluble and insoluble Aβ_42_ as well as p-tau181. Lipidomic profiling revealed accumulation of long-chain ceramides and sphingomyelins, with partial reversal of these effects upon inhibition of ceramide synthesis, implicating sphingolipid dysregulation as a mechanistic link between PFAS exposure and AD pathology. Supporting this, Huang et al. (2022) [36] used chimeric organoids mixing isogenic *APOE ε3* and *ε4* lines to show that astrocytic APOE4 drives lipid dysregulation while neuronal APOE4 increases Aβ, with both cell types required for robust tau pathology, findings that provide a mechanistic basis for the observed gene–environment synergies. To this end, Mousavi et al. (2025) [37] identified mitochondrial dysfunction and neuroinflammation as key events induced by PFAS in the pathogenesis of AD via an AOP network, suggesting that such environmental factors could be studied in 3D models to assess their impact on shared mechanisms.

Ji et al. (2025) [38] introduced a vascularized, neuroimmune organoid model that, when treated with AD patient brain extracts, developed plaques, tangles, microglial activation, synaptic loss, and network deficits, partially rescued by lecanemab, an anti-Aβ treatment. By incorporating patient-derived or engineered risk alleles, these models provide a platform where genetic susceptibility and environmental triggers can be studied alongside therapeutic interventions.

Importantly, converging human studies strengthen the rationale for these models. The *APOE ε4* allele is present in ~40–65% of AD patients, underscoring its role as a major genetic susceptibility factor [7]. PFAS have been directly detected in human cerebrospinal fluid, where their levels correlate with AD biomarkers and cognitive impairment [39]. Plasma PFAS also associate with altered apolipoprotein subspecies, including APOE [40], and serum biomonitoring revealed significantly higher PFAS burdens in AD compared with mild cognitive impairment and controls [41]. Together, these findings suggest that PFAS exposures may interact with apolipoprotein pathways, plausibly amplifying *APOE ε4*-related vulnerability in AD.

## 3. Parkinson’s Disease: Midbrain Organoids and Dopaminergic Models

Parkinson’s disease (PD) involves the degeneration of midbrain dopaminergic neurons and α-synuclein aggregation. Both genetic mutations (e.g., *LRRK2*, *SNCA*) and environmental toxins (pesticides, heavy metals, PFAS) contribute to risk. Epidemiological data from a case–control study in the U.S. found higher serum PFOS and PFOA levels in PD patients compared to matched controls, with an adjusted odds ratio of 1.8 per interquartile range increase in PFOS [42]. Experimentally, PFOS exposure induced selective dopaminergic neuron loss and motor deficits in zebrafish larvae [43] and PFOS has been shown to promote α-synuclein fibrillization in human SH-SY5Y cells [14].

Midbrain organoids derived from iPSCs produce functional dopaminergic neurons. Smits et al. (2019) [44] generated midbrain-like organoids from *LRRK2* p.G2019S patient iPSCs, which exhibited reduced dopaminergic neuron numbers and developmental defects. *SNCA*-triplication organoids show age-dependent α-synuclein aggregates and neuron loss. Co-cultures with microglia have demonstrated neuron–glia interactions leading to Lewy body-like pathology.

Tian et al. (2024) [45] leveraged a microfluidic-based midbrain organoid platform, embedding iPSC-derived dopaminergic organoids within a perfusable chip lined with human brain microvascular endothelial cells. They administered PFOS at 100 ng/mL continuously for 14 days to mimic chronic exposure. Using immunofluorescence for TH and Ki67, they quantified a 30% reduction in dopaminergic neuron proliferation and neurite outgrowth. Concurrent single-cell RNA-seq revealed upregulated inflammatory markers (IL-6, CXCL-10) in both astrocyte and microglia-like clusters, while multi-electrode array recordings showed a 40% decrease in spontaneous firing rate and network synchrony, indicating functional disruption. While midbrain organoids reveal how dopaminergic neuron loss impairs local network activity, in vivo and clinical evidence further demonstrates that PD-related circuit dysfunction extends to the cortico-subthalamic network. Recent electrophysiological studies indicate that aberrant synchronization within this network actively reshapes motor coding [46]. This model uniquely integrates vascular, glial, and neuronal elements to capture PFOS-driven neurodevelopmental toxicity in a human-relevant 3D microphysiological context. These findings support the role of PFAS in disrupting human dopaminergic development.

To bridge single-region organoids and circuit phenotypes in PD, nigrostriatal assembloids couple ventral midbrain and striatal tissues, reconstructing long-range dopaminergic projections, synaptogenesis, catecholamine handling, and early disease-like vulnerabilities [47]. These inter-regional constructs enable assays of axonopathies and α-synuclein propagation across connected targets, expanding beyond local midbrain readouts to a circuit-aware model. Practical constraints remain, assembly variability, limited throughput, and standardization, but this approach adds a connectivity-level lens not captured by single-region systems [47].

Gene–environment interactions can be further probed via isogenic CRISPR-corrected iPSC lines and single-cell transcriptomics highlighting convergent pathways (e.g., lysosomal, inflammatory) impacted by both genetics and toxins.

## 4. Multiple Sclerosis: 3D Glia-Enriched Models and Neuroimmune Interactions

Multiple sclerosis (MS) is an autoimmune demyelinating disease with strong genetic (*HLA-DRB1*) and environmental (EBV infection, vitamin D deficiency, smoking) factors. Epidemiological links between PFAS and MS onset remain inconclusive; earlier studies yielded null or inverse associations, while the recent Swedish longitudinal case–control study confirmed that hydroxylated PCBs increase multiple sclerosis (MS) risk but did not implicate per- and polyfluoroalkyl substances (PFAS) in disease initiation. Instead, several long-chain PFAS (PFOA, PFOS, perfluorodecanoic acid (PFDA), perfluoroheptane sulfonate (PFHpS), perfluorononanoic acid (PFNA)) were paradoxically associated with a reduced risk of disability worsening, particularly in males [48]. These findings suggest that PFAS may modulate immune responses in MS, yet the mechanisms remain unresolved and organoid models offer platforms to test such exposures.

Although in vitro models of NDDs are still incomplete, 3D culture methods offer an important new strategy to characterize disease mechanisms and facilitate the discovery of new therapies [49].

Fagiani et al. (2024) [50] developed a glia-enriched 3D organoid from MS patient iPSCs, showing impaired oligodendrocyte maturation and myelination deficits tied to low p21 expression. Daviaud et al. (2025) [51] described MS organoids carrying patient genetics, demonstrating intrinsic myelination failures independent of immune cells. These organoids can be challenged with triggers (EBV antigens, toxins, nutritional factors) to study gene–environment interactions.

BBB dysfunction and immune–endothelial interactions are critical in MS pathogenesis, with brain endothelial cells directly influencing T cell migration and neuroinflammation [52]. Adding microglia or peripheral immune cells on-chip may soon enable full “MS in a dish” models, elucidating how pollutants or infections precipitate demyelination in a genetically susceptible context.

A complementary human myelination platform integrates iPSC-derived neuronal and oligodendrocyte spheroids within a compartmentalized microfluidic device, where extending axonal fascicles become ensheathed by oligodendrocytes, recapitulating key features of human myelin biology and axon–glia coupling [53]. The system supports quantitative readouts (e.g., MBP-positive sheath counts/lengths) and targeted injury paradigms, offering a tractable setting to interrogate developmental myelination and repair with relevance to fetal/perinatal brain vulnerability. For PFAS studies, the controlled microenvironment and fluidic separation suit chronic, low-dose exposure designs, and complement established hiPSC neuron–oligodendrocyte co-cultures [54,55,56].

Future studies should emphasize prospective cohorts that track PFAS exposure alongside neuroinflammatory biomarkers, as well as comprehensive genotyping to uncover gene–environment interactions. Mechanistic experiments in 3D glia-enriched organoids are particularly needed to test PFAS impacts on oligodendrocyte maturation, neuroimmune responses, and myelination deficits. Incorporating immune cell components into organ-on-chip platforms will further clarify how pollutants or infections precipitate demyelination in genetically susceptible contexts. Advanced BBB organoid platforms composed of endothelial cells, pericytes, and astrocytes offer versatile tools for studying barrier permeability and immune infiltration under pathological conditions [57].

By integrating rigorous genotypic characterization with environmental exposure experiments in these human-relevant systems, researchers can disentangle genetic susceptibility from pollutant-driven effects in MS, ultimately clarifying whether PFAS act as risk modifiers or disease-course modulators.

**Figure 2:**
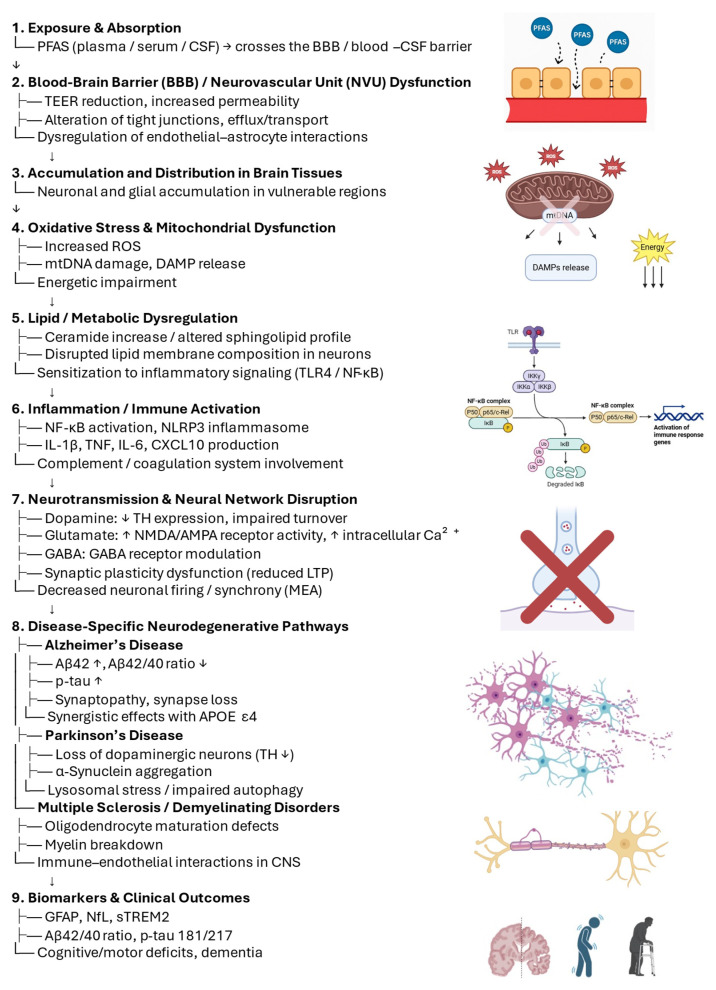
Converging mechanisms by which PFAS could influence neurodegeneration. Schematic of hypothesized links from exposure and BBB/neurovascular unit perturbation to brain accumulation, oxidative stress and mitochondrial dysfunction, lipid/ceramide dysregulation, and neuroinflammation (e.g., NF-κB, NLRP3), leading to neurotransmission and network disruption (dopamine, glutamate, GABA; reduced synaptic plasticity) and converging on AD/PD/MS hallmarks (Aβ/p-tau changes, dopaminergic neuron loss, myelination defects). Biomarker candidates include GFAP, NfL, sTREM2 and disease-specific markers such as Aβ42/40 and p-tau. Arrows indicate putative pathways; steps may occur in parallel with feedback loops.

The evidence reviewed in this section and in Section 2 and Section 3 highlights oxidative stress, mitochondrial dysfunction, lipid dysregulation, neuroinflammation, and synaptic impairments as converging mechanisms of PFAS-induced neurotoxicity across Alzheimer’s disease (AD), Parkinson’s disease (PD), and multiple sclerosis (MS). These processes are schematically integrated in Figure 2.

## 5. Leveraging 3D Models to Address Mechanistic Insights

Advanced 3D cellular platforms, such as patient-derived organoids and microphysiological systems, offer unique opportunities to dissect PFAS-driven neuropathological processes in a human-relevant context. By encapsulating genetic background, multicellular architecture, and long-term exposure paradigms, these models can clarify whether observed molecular changes (e.g., Aβ accumulation, tau hyperphosphorylation, ceramide-mediated inflammation) are true drivers of disease or secondary phenomena. Converging evidence suggests that network hyperactivity can emerge at the early stages of pathology, reinforcing the need for advanced in vitro platforms capable of capturing such functional alterations in a human-relevant context [58]. Representative systems and their applications are summarized in Table 1, including cerebral organoids carrying *APOE ε4* alleles that can be chronically exposed to environmentally relevant PFAS concentrations to determine direct effects on amyloidogenic processing and lipid dysregulation, while microfluidic midbrain chips allow for real-time monitoring of dopaminergic neuron viability and neuroinflammatory crosstalk under continuous PFAS perfusion. Combining these systems with CRISPR-engineered isogenic controls and high-content readouts (transcriptomics, lipidomics, electrophysiology) will enable a mechanistic hierarchy: identifying primary PFAS targets, mapping downstream signaling cascades, and testing interventions. Such an integrated 3D-model framework supersedes the need for reverse-causality exclusions in epidemiological designs by recreating key aspects of disease progression in vitro and directly probing causality at the cellular level. Traditional models, however, often lack human relevance or fail to reproduce the multicellular complexity of the central nervous system. Recent advances in stem cell biology and bioengineering have therefore enabled the development of next-generation platforms, which provide more physiologically relevant insights into PFAS-induced neurotoxicity.

### 5.1. Platform Selection for PFAS × Neurodegeneration

**Figure 3:**
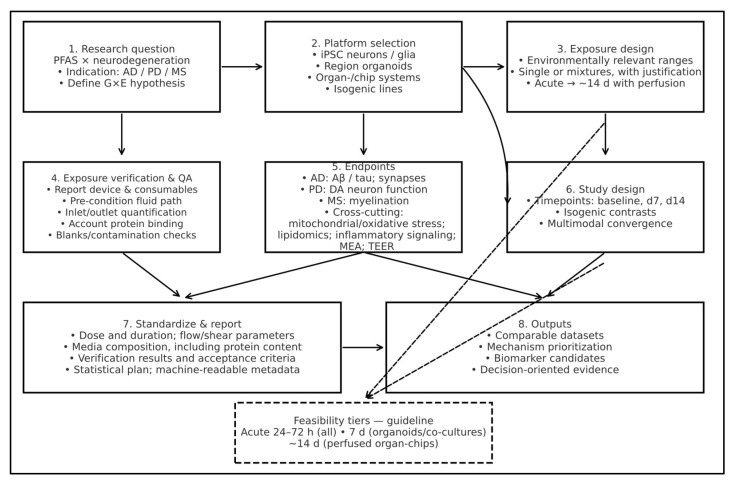
Feasibility roadmap for PFAS–neurodegeneration studies. (1) Define indication and G × E hypothesis; (2) select the minimal model (iPSC neurons/glia, region organoids, organ-on-chip, isogenic lines); (3) pre-specify exposure (environmental relevant ranges; single/mixtures; duration); (4) verify exposure and QA (materials disclosed, inlet/outlet mass balance, protein binding, blanks); (5) choose indication-aligned and cross-cutting endpoints; (6) set timepoints and isogenic contrasts with multimodal readouts; (7) standardize and report methods and metrics; (8) obtain comparable, mechanism-oriented outputs. Solid straight arrows indicate the primary workflow; solid curved arrows indicate ancillary dependencies (e.g., platform selection → study design; endpoints → outputs). Dashed arrows point to the dashed box, a feasibility guideline summarizing practicable exposure durations: 24–72 h across systems; ~7 d in organoids/co-cultures; ~14 d in perfused organ-chips and informs steps 3 and 6. Arrows denote information flow rather than causality. The workflow is iterative: QA or readouts can require refining exposure design, model choice, or timepoints.

Advanced human models should be matched to the mechanistic question and time scale. iPSC-derived neurons and glia resolve cell-intrinsic responses and enable isogenic gene–environment (G × E) contrasts. Region-specific organoids (cerebral, midbrain, glia-enriched MS organoids) capture multicellular phenotypes—Aβ/tau and synaptic changes (AD), dopaminergic vulnerability and network activity (PD), oligodendrocyte maturation and myelination (MS)—with electrophysiology and multi-omics endpoints. Microphysiolog-ical/organ-on-chip systems add controlled flow and barrier modules when exposure physiology is key, supporting readouts such as TEER and transport. CRISPR-engineered isogenic lines (including base/prime editing) isolate allele effects (e.g., *APOE ε4*, *LRRK2*, HLA-DRB1*15:01) across identical exposures. Multi-compartment configurations (barrier → parenchyma) enable exposure-to-effect workflows where relevant. Aligned with our roadmap (Figure 3), we advocate a barrier-first, escalation-next execution to quantify G × E interactions: first parameterise exposure at the blood–brain barrier using BBB organoids or BBB-on-chip (TEER, permeability/transport, efflux/transporter function under shear, with inlet–outlet mass-balance verification), then escalate to advanced 3D human systems matched to indication. For AD, vascularised, neuroimmune cerebral organoids—including microglia-competent variants—interrogate the interplay between lipid handling, amyloid/tau processing and neuroinflammation; for PD, nigrostriatal assembloids recover long-range dopaminergic connectivity and enable assays of axonopathy and α-synuclein propagation; for MS, human iPSC myelination co-cultures and emerging microfluidic myelination devices provide quantitative, human-specific myelin formation and repair endpoints. Across models, exposures should be chronic, low-dose and mixture-aware (anchored to biomonitoring distributions), genotype contrasts pre-specified (e.g., *APOE ε4*, *LRRK2*, HLA-DRB1*15:01), and read-outs harmonised to cross-platform biomarker panels (GFAP, NfL, sTREM2). Table 1 operationalises this pathway by reporting, for each platform, core read-outs together with PFAS-exposure status and representative genetic backgrounds. Selection should be paired with explicit exposure verification (reported device materials, inlet/outlet quantification, protein binding, blanks) and standardized reporting to support cross-study comparability.

Figure 3 summarizes an operational sequence for designing and reporting experiments that test PFAS–neurodegeneration hypotheses in advanced cellular systems. Investigators should pre-specify the indication and G × E question, then select the minimal model that captures the required biology. Exposure conditions are defined a priori with environmentally relevant concentrations, justified single congeners and/or mixtures, and durations compatible with platform viability (acute to ~7 days in static or semi-static formats; up to ~14 days with perfusion). A mandatory exposure verification & QA step ensures that nominal doses translate into effective dosing: transparently report device/consumable materials; pre-condition the fluid path; quantify inlet/outlet concentrations to establish mass balance and estimate free concentrations; account for protein binding and potential carryover; include blanks/contamination checks; and set acceptance criteria before outcome analysis. Endpoints should map to indication-specific biology (e.g., AD: Aβ/tau and synaptic measures; PD: dopaminergic function; MS: myelination) alongside cross-cutting metrics (mitochondrial/oxidative stress, lipidomics, inflammatory signaling, barrier integrity where applicable, and network activity via multielectrode array (MEA). Study architecture incorporates predefined timepoints (e.g., baseline, day 7, day 14), isogenic contrasts, randomization/blocking, viability monitoring, and a pre-specified analysis plan (effect sizes, multiplicity control, batch correction). Reporting includes nominal and measured exposure metrics, media composition (including protein content influencing PFAS availability), flow/shear parameters when relevant, verification results, and machine-readable metadata. Implemented in this way, the workflow supports mechanism-focused inference and cross-study synthesis.

Notwithstanding these strengths, interpretation should account for indication-specific and practical constraints of each platform.

Current platforms have indication-specific constraints that shape interpretation. Cerebral organoids often lack vascularization, show incomplete neuronal maturation, and do not include fully functional microglia; in several reports the PFAS ranges are not physiological, which can distort effect size and relevance. Midbrain organoid and chip systems show reduced neuronal maturation, a simplified microenvironment, short feasible exposure windows, and no long-range circuit connectivity, limiting extrapolation to systems-level PD phenotypes. Glia-enriched MS organoids provide targeted myelination readouts but lack a complete immune system, have limited neuronal development, and miss peripheral modulators of myelination, constraining neuroimmune conclusions. Cross-cutting issues include incomplete maturation across models and practical limits on long-term exposure, underscoring the need for explicit exposure verification and complementary use with other model types.

### 5.2. Extending Model Applicability to Other Environmental Risk Factors

In addition to per- and polyfluoroalkyl substances, the advanced human cellular platforms described above can be broadly applied to investigate diverse environmental risk factors for neurodegeneration. Induced pluripotent stem cell (iPSC)–derived neurons and glia, three-dimensional brain organoids, and microphysiological systems (e.g., blood–brain barrier chips) enable mechanistic dissection of how toxicants such as pesticides, heavy metals, air pollutants, and viral antigens contribute to neural dysfunction. Each of these exposure classes is linked to neuropathogenic processes, ranging from mitochondrial injury and oxidative stress to protein aggregation and neuroinflammation, that mirror pathways seen in idiopathic neurodegenerative diseases. Below, we highlight how state-of-the-art in vitro models can recapitulate these mechanisms for each toxicant class, and how combining patient-derived cells with environmental challenges can illuminate gene–environment interactions underlying disorders like AD, PD, and MS.

Pesticides: A large body of epidemiological and experimental evidence implicates chronic pesticide exposure in the pathogenesis of PD [60]. Many widely used insecticides and herbicides (for example, organophosphates, paraquat, and rotenone) inflict oxidative stress and mitochondrial dysfunction in neurons, accelerating α-synuclein misfolding and dopaminergic cell death characteristic of PD [61]. Human iPSC-based models have proven especially useful for pinpointing such pesticide effects. In a recent study, midbrain dopaminergic neurons derived from PD patient iPSCs were screened against dozens of pesticides identified in epidemiologic analyses; this in vitro platform confirmed that at least 10 pesticides directly trigger dopaminergic neurotoxicity, with one herbicide (trifluralin) found to induce pronounced mitochondrial impairment [60]. Notably, combinations of pesticides produced greater neuronal damage than any single compound, underscoring the polychemical exposures typical in agriculture [60]. Three-dimensional midbrain organoids, which self-organize dopaminergic neurons with astrocytes, may provide another advantageous system to observe pesticide-induced nigrostriatal degeneration in a human context. Because these models can be generated from patient-specific iPSCs, they also permit gene–environment interaction studies. For example, individuals carrying certain polymorphisms in the detoxifying enzyme PON1 show a >2-fold higher PD risk upon organophosphate exposure [62]. By deriving neurons or organoids from such genetically susceptible donors, researchers can directly examine how impaired pesticide metabolism or other genetic liabilities (e.g., variants in dopamine transport or vesicular storage [62]) exacerbate neurotoxic outcomes, thus recapitulating the synergistic effect of pesticides and PD risk genes observed in population studies. Similarly, organochlorine pesticides and related herbicides correlate with elevated MS incidence in agricultural populations [63].

Heavy Metals: Toxic metal exposures (lead, mercury, cadmium, aluminum, manganese, and others) are long-recognized environmental contributors to cognitive decline and neurodegenerative disease. Heavy metals can disrupt metal homeostasis in the brain and promote the misfolding and aggregation of key proteins. For instance, lead exposure up-regulates APP and β-secretase, increasing Aβ production; aluminum and cadmium can bind directly to Aβ peptides, fostering plaque formation [64]. Excess metals also catalyze redox reactions that generate reactive oxygen species, compounding oxidative neuronal damage [64]. In parallel, metal neurotoxicity often involves a strong neuroinflammatory component: heavy metal exposure activates microglia and astrocytes, driving up production of proinflammatory cytokines (IL-1β, TNF-α, IL-6, etc.) that can chronically injure neurons [64]. Advanced in vitro systems are uniquely poised to capture these multicellular interactions. Cerebral organoids containing mixed neuronal and glial populations can model how a metal like cadmium simultaneously induces Aβ aggregation and glial-mediated inflammation in human neural tissue, something difficult to reproduce in animal models [64]. Likewise, dynamic microfluidic models of the blood–brain barrier (BBB) offer insight into metal translocation and barrier dysfunction, for example, demonstrating that a compromised BBB permits greater lead ingress into the brain parenchyma, thereby heightening neurotoxic effects [65]. Crucially, iPSC-based models allow investigators to incorporate human genetic risk factors to probe metal–gene interactions. *APOE ε4* appears to modulate the brain’s response to metals; one clinical study found that *APOE ε4* carriers suffered more pronounced cognitive impacts from elevated manganese and zinc exposure than did non-carriers [66]. Using isogenic iPSC lines differing only by *APOE ε* genotype (e.g., *APOE ε3/ε4* vs *APOE ε3/ε3*, etc.), researchers can generate otherwise identical neural cultures or organoids and compare their vulnerability to metal-induced oxidative stress and inflammation. Early findings indeed suggest that an *APOE ε4* background exacerbates metal accumulation and inflammatory signaling in neurons and glia, providing a human-based demonstration of gene–environment synergy in metal neurotoxicity. Epidemiological studies have linked mercury and lead exposure to higher MS risk, with animal models showing that low-dose mercury accelerates autoimmunity and lead induces T-cell dysfunction and oxidative stress. A 3D human iPSC-derived model containing oligodendrocytes, astrocytes, and microglia could significantly enhance mechanistic understanding of how such metals compromise myelin integrity and trigger immune-mediated demyelination [67].

Air Pollution: Airborne pollutants, especially fine particulate matter (PM_2.5_ and smaller), traffic-derived combustion aerosols, and associated toxic gases, are increasingly recognized as modifiable risk factors for neurodegenerative diseases [68]. Chronic inhalation of polluted air is epidemiologically linked to accelerated cognitive aging and AD pathology, likely via multifactorial mechanisms. Particulates and ultrafine particles can translocate into the brain and instigate oxidative stress, perturb mitochondrial function, and promote protein aggregation in neural cells [61,68]. Perhaps most critically, air pollution induces robust neuroinflammation: particulate irritants activate peripheral immune responses that in turn trigger microglial activation behind the BBB, establishing a feed-forward cycle of CNS inflammation. To unravel these processes under controlled conditions, researchers have turned to microphysiological models that integrate a human BBB with 3D brain tissue. In one recent advance, a “brain-on-chip” platform was used to expose neural cultures to actual urban PM_2.5_ under flow. This model elegantly recapitulated the sequence of events by which inhaled particulates may drive neurodegeneration [69]. The investigators demonstrated that fine particles crossed the BBB-on-chip and deposited into the brain compartment, where they stimulated astrocytes to become reactive (astrogliosis) and induced the infiltration and M1 polarization of microglia [69]. The cytokines and nitric oxide released by activated glia in turn inflicted synaptic damage, provoked tau hyperphosphorylation, and caused frank neuronal loss, pathological changes analogous to those seen in early AD [69]. Such findings underscore how air pollution can hasten neurodegenerative cascades predominantly through neuroimmune mechanisms. Notably, the deleterious effects of pollution may be heightened in genetically at-risk individuals. *APOE ε4* carriers, for example, appear particularly sensitive to air pollution–related brain atrophy: older women with *APOE ε4* who lived near dense traffic exhibited significantly greater hippocampal volume loss than *APOE ε4* non-carriers in the same environment [70]. This gene–environment interaction is being explored in vitro by differentiating *APOE ε4* vs. *APOE ε3* iPSC-derived brain cells and subjecting them to diesel exhaust or particulate exposure, to observe genotype-dependent differences in neuroinflammatory responses. Combining patient-specific organoids with controlled pollutant exposures will thus be key to predicting which populations are most vulnerable to pollution-induced neurodegeneration and why. Recent cohort analyses also implicate persistent organic pollutants, for example, hydroxylated PCB metabolites, in increased MS onset risk [48]. Air pollution, including fine particulate matter and polycyclic aromatic hydrocarbons, is increasingly recognized as synergizing with genetic predisposition to drive neuroinflammation and MS onset [71].

Viral Antigens: Infectious pathogens, particularly neurotropic viruses, have long been suspected as environmental triggers that can initiate or exacerbate neurodegenerative processes. Viruses such as herpes simplex virus type 1 (HSV-1) can establish lifelong latent infections in the brain and are postulated to contribute to amyloid plaque deposition and neuroinflammation in AD [72]. The proposed mechanism involves periodic reactivation of the virus in the CNS, which elicits an innate immune response (microglial and astrocytic activation) and the release of neurotoxic inflammatory mediators; these in turn may promote neuronal damage and abnormal protein aggregation (e.g., HSV-1 infection has been shown to induce accumulation of beta-amyloid and hyperphosphorylated tau in infected neural cultures) [72]. Advanced in vitro models are uniquely suited to interrogate these host–pathogen interactions in human neural tissue. Three-dimensional “neuroimmune” organoids that include microglia-like cells have been used to model viral exposure in the brain. Notably, when human brain organoids or bioengineered neural tissues were infected with HSV-1, they developed striking Alzheimer-like pathology: extracellular amyloid plaque–like deposits surrounded by infected cells, widespread gliosis, and functional synaptic deficits, all arising in the absence of any familial AD mutations [72]. This experimental infection paradigm strongly supports a causal link between viral antigen exposure and AD-like neurodegeneration, especially when specific host genetic risk factors are present [72]. Indeed, organoids carrying the *APOE ε4* allele exhibited exacerbated neuronal dysfunction and inflammation upon HSV-1 infection, mirroring clinical data that HSV-1 positive *APOE ε4* carriers are at heightened risk of Alzheimer’s dementia [72]. Similar synergy may apply to other viruses: for example, *APOE ε4* genotype is associated with increased blood–brain barrier permeability, which can facilitate the entry of circulating viruses (such as coronaviruses) into the brain and thereby amplify neuroinflammatory injury [73]. Going forward, BBB-on-chip systems seeded with human endothelial cells and astrocytes can be inoculated with neurotropic viruses or viral proteins to study barrier transmigration and ensuing neuroimmune reactions in real time. By leveraging patient-derived cell models with defined genetic backgrounds, scientists can examine how host factors like *APOE ε4*, *TREM2*, or *SNCA* mutations modulate the brain’s vulnerability to viral challenges. Such platforms will be invaluable for testing antiviral or immunomodulatory interventions aimed at interrupting the hypothesized microbe-induced acceleration of neurodegenerative disease. In parallel, virtually all MS patients have been infected with EBV, and meta-analyses show EBV seropositivity alone confers ~2.6-fold MS risk, which is further amplified in HLA-DRB1*15:01 carriers [74]. Recent studies using human BBB microphysiological systems have demonstrated that exposure to inflammatory cytokines, mimicking MS lesions, increases permeability in neurovascular-unit organoids comprising neurons, oligodendrocytes, astrocytes, microglia, endothelial cells, and pericytes. Building on this work, multicellular 3D neurovascular-unit organoids generated from human iPSC-derived brain microvascular endothelial cells together with pericytes, astrocytes, microglia, oligodendrocytes, and neurons provide a human-relevant platform to interrogate how hypoxia and neuroinflammatory cues jointly disrupt BBB structure and function [59].

## 6. Conclusions

This perspective highlights how advanced human-based platforms—ranging from iPSC-derived neuronal and glial cultures to brain region-specific organoids and organ-on-chip systems—are transforming neurotoxicology. Initially developed to disentangle the neurotoxic potential of PFAS and their mechanistic links with AD, PD, and MS, these models now offer a broader translational framework to explore the neurobiological impact of diverse environmental exposures.

By extending their use to pesticides, metals, airborne pollutants, and viral agents, researchers can uncover both converging and exposure-specific pathways that underlie sporadic neurodegenerative disease. Such models uniquely enable experimental control over both the environmental insult and the genetic background of neural tissue, thus capturing the complex gene–environment interplay that drives vulnerability to brain aging.

As shown across different classes of toxicants, these systems reveal common mechanisms—mitochondrial dysfunction, oxidative stress, protein aggregation, disruption of the blood–brain barrier, and chronic neuroinflammation—that represent shared final routes of environmental neurodegeneration.

Beyond mechanistic insight, this integrative approach bridges epidemiological associations with causal understanding and opens the way for human-relevant screening of preventive strategies, including antioxidant, anti-inflammatory, or metabolic interventions.

Ultimately, the convergence of next-generation in vitro models with omics-based and computational analyses will strengthen the predictive capacity of neurotoxicology, improve the identification of high-risk environmental exposures, and support the development of actionable strategies to mitigate their contribution to neurodegenerative disorders of aging.

## Figures and Tables

**Table 1:** 3D cellular models available for PFAS neurotoxicity. This table summarises model types (organoids, assembloids and microphysiological systems), disease indication, core read-outs, strengths/limitations, genetic background, and PFAS exposure status.

Model	Indication	Core Readouts	Distinct Strengths	Key Limitations	Genetic Background	Other Risk Factor(s)	PFAS Exposure	Representative Refs
Cerebral organoids (human iPSC-derived)	AD	Aβ42/40, p-tau (e.g., p-tau181/217), lipidomics (ceramides), synaptic/network activity	Human genetics; spontaneous AD-like phenotypes; compatible with chronic PFAS exposure	Limited vascularization and microglia; maturation variability across protocols	*APOE ε3/ε4*; familial *APP*/*PSEN1*/*PSEN2*; isogenic WT controls	pesticides; heavy metals; persistent organic pollutants (POPs; PCBs, dioxins); fine/ultrafine air pollution	Yes—PFOA, PFOS, PFHxS ([31])	[31,33]
Vascularized neuroimmune cerebral organoids	AD	Plaque-/tangle-like pathology, microglial activation, synapse loss, network impairments; rescue by therapeutics	Integrates vasculature + immune components; reproduces multiple AD hallmarks	Protocol complexity; exposure paradigms still consolidating	WT hiPSC backgrounds; optionally AD-risk (e.g., *APOE ε4*) lines	fine/ultrafine air pollution; metal mixtures; POPs; viral antigens/neurotropic viruses	Not reported in cited refs	[39]
Chimeric cerebral organoids mixing *APOE ε3/ε4* cells	AD	Astrocytic lipid dysregulation, neuronal Aβ increase, robust tau pathology when both lineages carry *APOE ε4*	Dissects cell-type–specific *APOE ε4* contributions; human genetic context; aligns with lipid–amyloid–tau axis	Batch variability; requires careful cell-mixing ratios; limited vasculature unless engineered	*APOE ε3* vs. *APOE ε4* mixed chimeras; isogenic backgrounds	heavy metals; air pollution; pesticides; POPs (for *APOE*–environment interactions)	Not reported in cited refs	[36]
Isogenic CRISPR-edited AD organoids (e.g., *APOE ε4* knock-in; *PSEN1/2* familial mutations)	AD	Genotype-controlled Aβ42/40 shifts, p-tau species, synaptic and network phenotypes; multi-omics contrasts	Clean G × E contrasts with isogenic backgrounds; mechanistic attribution to single alleles	Editing/clone variability; maturation time and batch effects; rigorous QC of edits/off-targets required	*APOE ε4*; *PSEN1/2*; *APP* (Swedish; KM670/671NL); isogenic WT controls	pesticides; heavy metals; air pollution; POPs; viral antigens (for allele-specific G × E studies)	Not reported in cited refs	[32,34]
3D human brain-like tissue model triggering AD-like pathology via viral challenge	AD	Aβ accumulation, neuronal loss; scaffold-based 3D readouts with immunostaining and functional assays	Demonstrates environmental/triggered induction of AD-like features in human 3D tissue	Model depends on specific triggers; not yet standardized for PFAS exposures	Typically WT donor lines	viral antigens/infections (HSV-1, SARS-CoV-2 surrogates); virus + pollutant co-exposures	Not reported in cited refs	[35]
Coupled BBB → cerebral organoid microphysiological systems (two-compartment perfusion)	AD (exposure interface)	TEER, PFAS partitioning/translocation, endothelial–astrocyte crosstalk, downstream neuronal/glial responses	Physiologic delivery under flow; quantitative mass-balance of exposure; barrier injury readouts	Device material adsorption; fluidic complexity; multicomponent QA needed for dose verification	Barrier: generic hiPSC endothelium; Parenchyma: organoid genetics as specified (e.g., *APOE ε4*, *PSEN1*)	soluble air-pollution components; heavy metals; pesticides; POPs; circulating viral antigens	Not reported in cited refs	[57]
BBB organoids/BBB-on-a-chip	Cross-disease exposure interface	TEER, permeability/transport, efflux/transporter function, cytokines under shear	Human barrier biology; mass-balance dosing verification; physiological shear and flow	Requires materials disclosure/adsorption control; neurovascular-immune complexity limited unless co-cultured	Generic hiPSC lines or hCMEC-like endothelium; isogenic NVU co-cultures optional	air pollution (BBB-focused effects); heavy metals; pesticides; small organic toxicants; viral antigens.	Not reported in cited refs (platform suited for PFAS perfusion)	[52]
Multicellular 3D neurovascular-unit organoids (NVU-like)	Cross-disease neurovascular interface (hypoxia/neuroinflammation/BBB dysfunction; relevant to MS, stroke and neurodegeneration)	Macromolecular permeability (albumin, IgG, FITC–dextran), tight-junction and adherens markers (ZO-1, occludin, claudin-5, VE-cadherin), BBB transporters (e.g., MDR-1, AQP4, GLUT-1), basement membrane proteins (fibronectin, laminin, collagen IV), oxidative stress and ATP levels, inflammatory and chemotactic cytokines (e.g., IL-1β, IL-6, IL-8, TNF-α, MCP-1)	Fully human multicellular NVU spheroids integrating brain microvascular endothelial cells, pericytes, astrocytes, microglia, oligodendrocytes and neurons; recapitulate hypoxia- and cytokine-induced BBB breakdown, oxidative stress and cytokine “storms”; demonstrated suitability for testing anti-inflammatory/antioxidant compounds and conceptually well suited to evaluate environmental toxicants	Static organoid format without defined luminal/abluminal perfusion or physiological shear; diffusion-limited exposure gradients and size heterogeneity; no adaptive immune cells (T/B lymphocytes); original implementation uses generic donor cells rather than patient-specific or isogenic lines; environmental chemicals (including PFAS) not yet tested	Human brain microvascular endothelial cells combined with human pericytes, astrocytes, microglia, oligodendrocytes and neurons; generic human donor backgrounds (not isogenic in the original study)	heavy metals; air pollution (PM_2.5_, ultrafine particles); POPs; viral infections (neurovascular/MS-relevant)	Not reported in cited refs (platform technically suited for PFAS perfusion or static exposure, including under hypoxic/inflammatory conditions)	[59]
Glia-enriched human brain organoids (patient iPSC)	MS	Oligodendrocyte maturation (MBP/MAG/PLP), myelination deficits, glial-immune phenotypes	Human background; direct readouts of oligodendrocyte dysfunction	Reduced neuronal complexity; immune system not complete	Patient-derived (e.g., RRMS/SPMS) and matched controls	heavy metals; pesticides; POPs; particulate air pollution; viral/inflammatory MS triggers (e.g., EBV mimetics)	Not reported in cited refs	[50,51]
Human iPSC myelination co-cultures/microfluidic myelination chips	MS	De novo myelin formation (MBP + sheaths), sheath length/number; injury/repair assays	Quantifiable human myelination endpoints; suited for remyelination screens	Technically demanding; typically lacks full immune context	WT donor lines; disease-specific (e.g., PMD/MLC) lines in some studies; isogenic edits possible	heavy metals; pesticides; POPs; air pollution; viral/inflammatory cues impairing myelin repair	Not reported in cited refs	[54,55,56]
Midbrain organoid microphysiological system (perfusion chip)	PD	TH+ neuron viability, neurite outgrowth, MEA activity, scRNA-seq inflammatory signatures	Dopaminergic specificity; controlled flow and exposure; quantitative functional endpoints	Short feasible exposure windows; simplified niche; limited long-range connectivity	*LRRK2* (e.g., p.G2019S) or WT; optional *SNCA* overexpression	PD-linked pesticides (paraquat, rotenone, etc.); manganese and other dopaminergic metals; combustion-derived particles	Yes—PFOS ([46])	[46]
Nigrostriatal assembloids (midbrain–striatum; MISCO variants)	PD	DA axonal projections, synaptogenesis, catecholamine release, α-syn propagation	Models’ long-range connectivity missing in single-region organoids	Assembly adds variability; limited throughput and standardization	Age-induced in WT lines; PD-mutant variants possible	PD-linked pesticides; manganese and related metals; air-pollution-derived toxicants driving nigrostriatal axonopathy	Not reported in cited refs	[47]

## Data Availability

The original contributions presented in the study are included in the article, further inquiries can be directed to the corresponding authors.
